# Understanding Gaps Between Daily Living and Clinical Settings in Chronic Disease Management: Qualitative Study

**DOI:** 10.2196/17590

**Published:** 2021-02-25

**Authors:** Mustafa Ozkaynak, Rupa Valdez, Katia Hannah, Gina Woodhouse, Patrick Klem

**Affiliations:** 1 College of Nursing University of Colorado | Anschutz Medical Campus Aurora, CO United States; 2 Department of Public Health Sciences University of Virginia Charlottesville, VA United States; 3 University of Colorado Hospital Aurora, CO United States

**Keywords:** health information systems, workflow, self-management, activities of daily living, mobile phone

## Abstract

**Background:**

Management of chronic conditions entails numerous activities in both clinical and daily living settings. Activities across these settings interact, creating a high potential for a gap to occur if there is an inconsistency or disconnect between controlled clinical settings and complex daily living environments.

**Objective:**

The aim of this study is to characterize gaps (from the patient’s perspective) between health-related activities across home-based and clinical settings using anticoagulation treatment as an example. The causes, consequences, and mitigation strategies (reported by patients) were identified to understand these gaps. We conceptualized gaps as latent phenomena (ie, a break in continuity).

**Methods:**

Patients (n=39) and providers (n=4) from the anticoagulation clinic of an urban, western mountain health care system were recruited. Data were collected through primary interviews with patients, patient journaling with tablet computers, exit interviews with patients, and provider interviews. Data were analyzed qualitatively using a theory-driven approach and framework method of analysis.

**Results:**

The causes of gaps included clinician recommendations not fitting into patients’ daily routines, recommendations not fitting into patients’ living contexts, and information not transferred across settings. The consequences of these gaps included increased cognitive and physical workload on the patient, poor patient satisfaction, and compromised adherence to the therapy plan. We identified resources and strategies used to overcome these consequences as patient-generated strategies, routines, collaborative management, social environment, and tools and technologies.

**Conclusions:**

Understanding gaps, their consequences, and mitigating strategies can lead to the development of interventions that help narrow these gaps. Such interventions could take the form of collaborative health information technologies, novel patient and clinician education initiatives, and programs that strongly integrate health systems and community resources. Current technologies are insufficient to narrow the gaps between clinical and daily living settings due to the limited number and types of routines that are tracked.

## Introduction

### Background

For patients with chronic conditions, health management is a continuous effort [1]. Management of chronic conditions entails numerous activities in both clinical (eg, developing plans of care) and daily living settings (eg, assessing medication side effects, controlling food intake) [2]. Activities across these settings interact [3-5], creating a high potential for a *gap* to occur if there is an inconsistency or disconnect between controlled clinical settings and complex daily living environments. We conceptualized *gap* as *a break in continuity in the performance of health-related activities across clinical and daily living settings*. A gap between health-related activities across settings is a latent phenomenon that can be identified or addressed only after precursors have surfaced or effects have been manifested (eg, when suboptimal health outcomes have occurred). As an example of a gap [4], medication prescription is a part of the clinical workflow that requires coordination with the patient’s daily living environment. Not incorporating daily living information to tailor therapy could lead to nonadherence. The study by Ozkaynak et al [4] provides more information on the definition of a gap. Identifying the specific points where a health care regimen can or must be modified is possible only if all health-related activities in both settings are considered part of a health care continuum [4-8]. Adding a gap construct to known models and frameworks may allow for a better comparison between theoretical and real-world management systems for patients.

This study aims to understand why these gaps occurred, what effects they produced, and preventative or remedial actions. The work was guided by the *Infinicare* framework [4], a theoretical perspective that highlights the need for integration of health-related activities across care delivery settings and accounts for patient-specific contextual features. The *Infinicare* framework has 3 principles. First, clinic-based health-related activities shape or influence activities in the context of daily living. Second, health-related activities within daily life inform activities in the context of the clinical setting [4]. Third, the *Infinicare* framework highlights the importance of context and operationalizes it by incorporating 4 different contextual dimensions: physical, organizational, social, and cultural. These 4 dimensions of context interdependently affect health-related activities in both clinical and daily living settings. The *Infinicare* framework posits that health and health care have no strict temporal or physical boundaries.

### Literature Review

#### Connecting Fragmented Health Care Services for Continuity of Care

Traditional studies of fragmentation within health care delivery and continuity of care focus exclusively on the clinical setting and demonstrate the adverse consequences that result from a failure either to defragment or to establish continuity of health care services [9,10]. Interventions (eg, secure messaging, mHealth, communication skill building) designed to address gaps across clinical settings have improved outcomes [11-14]. Our work seeks to expand this paradigm by understanding latent gaps through precursors and consequences that occur *between* clinical and daily living settings. Approaches such as patient-oriented workflow [15], patient journey [16], and adherence work [17] posit that health-related activities in the clinical and daily living settings are parts of a broader workflow. Research connecting clinical and home settings has focused on postacute care as related to hospital discharge. Gaps that occurred after hospital discharge (for both acute and chronic conditions) have focused on adverse drug events, avoidable hospitalizations, and medical errors [18,19]. A limited number of studies have examined the interplay between clinical care and self-management in the daily living environment [3,17,20]. Identifying factors that contribute to gaps [18] can inform interventions such as standardized communication protocols [21], secure messaging [22], personal health records [1], medication reconciliation strategies [23], patient and family education [24], community-based support [24], and patient navigators [25].

Interventions for continuity of care across settings have been institution centric [26]. These interventions (eg, discharge planning, home-visiting programs) are not necessarily tailored to an individual’s unique daily living circumstances, which obviates a thorough understanding of how these interventions may affect therapy and patient outcomes. Moreover, being institution centric, these interventions focus on the clinician rather than emphasizing patient-clinician collaboration as the driver of optimal outcomes [27,28]. Models and frameworks to describe daily living–related barriers to adherence have been developed (eg, patient work system [2]).

The study of gaps across settings is more challenging, as it requires the application of methods for studying continuity of care in clinical to daily living settings, the latter having more variability [5,29]. Variabilities include individual characteristics (eg, preferences, values), physical environment, social environment, cultural factors, and organizational (eg, community) resources. A study of older adult patients with heart failure applied a *work system* to patient-performed work in self-management. This study highlighted the interaction between systemic components (eg, tasks, technology) in which the patient was embedded [2]. However, more research is needed to examine differences in how the patient work system is conceptualized to pinpoint gaps, the consequences of gaps, and strategies for addressing these areas.

#### Collaborative Health Information Technologies

We define collaborative health information technology (HIT) as an information technology (IT) that has a full functional interface with clinicians, patients, and all other care partners. Collaborative HIT refers to a combination of connected technologies (eg, personal health records, electronic health records [EHRs], mHealth apps, social media) or an ecosystem [30]. The use of collaborative HIT can potentially provide business value, socio-organizational value, technical process, and evolution value [31].

Collaborative HIT [32] is underdeveloped in terms of facilitating education, integrating resources, and allowing for information exchange [30]. For example, provider access to patient-generated data and patient access to personal health information are limited. This study is timely in that isolating and capturing gaps in health care delivery will inform the design of collaborative HIT for use by both patients and clinicians.

Most informatics tool-design models begin with a needs assessment [33,34]. To our knowledge, previous systematic, large-scale, qualitative needs assessments for health IT design have focused on either clinical activities or supporting health-related activities in daily life, not the space or gaps between these areas [35,36]. Consequently, the purpose of this study is to characterize gaps between clinical and health-related activities in daily life, using anticoagulation treatment as an example. This study is timely given that collaborative HIT is in its very early stages of development [30].

The contributions of this study include the following: a systematic exploration of reasons for gaps, consequences of gaps between diverse settings, strategies for stopping these gaps via a systems framework, and a patient-oriented workflow approach. Study findings have the potential to influence how existing interventions could be reimagined or expanded through design recommendations for collaborative health IT [37-39].

### Case Exemplar

Anticoagulation therapy with warfarin is a particularly rich example for understanding gaps between clinics and daily life [7]. It requires strict adherence by patients and collaboration with providers, as frequent dosing adjustments are often necessary [40]. Food and alcohol affect dosing; therefore, a strict limitation of foods high in vitamin K and limiting alcohol intake is required. Moreover, contextual factors such as family support, ability to purchase food, and avoidance of specific foods that can affect therapy must be considered. Monitoring the therapeutic dose of warfarin is achieved by measuring blood levels for clotting. The international normalized ratio (INR) is a measure that evaluates the therapeutic range of anticoagulation [41]. Patients require testing in the clinic setting as often as several times a week, as home anticoagulant monitoring was not available at the site studied. Warfarin or Coumadin interacts with common antibiotics; therefore, communication is essential by the patient with their non–clinic-based providers (eg, dentist, podiatrist) ordering an antibiotic. Good communication between the provider and patient about testing results and dosing decisions is crucial. A wrong dose can result in serious bleeding, thromboembolic events, and medical complications from the underlying medical problem for which the anticoagulant was ordered. Examining gaps in optimal treatment can reveal clinician, patient, and system-related factors leading to suboptimal dosing and inform interventions.

We recognize that the dynamics of other chronic conditions (eg, diabetes, HIV) may differ from anticoagulation management. The findings and design guidelines based on anticoagulation, however, have the potential to be translated where HIT supports self-management activities in individualized therapy plans across patient conditions.

## Methods

### Study Design

We used a qualitative study design to examine and characterize gaps between health-related activities across diverse settings. Qualitative design allows for exploring and gathering rich descriptions of the phenomena of interest (ie, gap) in a context. Data were collected from patients and providers (clinical pharmacists) in a single academic, hospital-based anticoagulation clinic through interviews and journaling. Data were analyzed qualitatively. This study was approved by the Colorado Multiple Institutional Review Board.

The *Infinicare* framework [4] shaped this study in 2 ways: (1) it guided the development of the data collection tools by ensuring that data were collected on basic components (eg, health-related activities in daily living and clinical settings and social, organizational, physical, and cultural context); and (2) guided data analysis through use of select *Infinicare* constructs (eg, daily routines, challenges of self-management, clinicians’ understanding of the patient’s daily living environment) for the initial codes.

### Settings and Sample

Patients were recruited from the anticoagulation clinic of an urban, western mountain health system in the United States. The clinic provides outpatient care and is staffed by 4 clinical pharmacists. The primary medication used by the clinic was warfarin, an anticoagulant in pill form. Most clinic visits were face-to-face; however, phone visits also took place.

Exclusion criteria were individuals younger than 20 years of age. In an effort to approximate a real-world setting, participants with comorbid medical conditions, mental health conditions, substance use disorders, or pregnancy were eligible for inclusion. We used a stratified sampling strategy to ensure that we recruited older adult patients as well as younger patients of both genders and those both new and experienced with anticoagulation therapy. One participant’s first language was Spanish, for which a bilingual translator was used for data collection.

### Recruitment

Fliers were posted in the clinic with information about the objective of the study, sponsors, principal investigator, institutional review board approval number, and contact information for the research team. Interested participants called the dedicated phone number to provide their contact information. This information was accessed only by the research team, who called to verify that the prospective participant met the inclusion criteria.

### Data Collection

**Figure 1:**
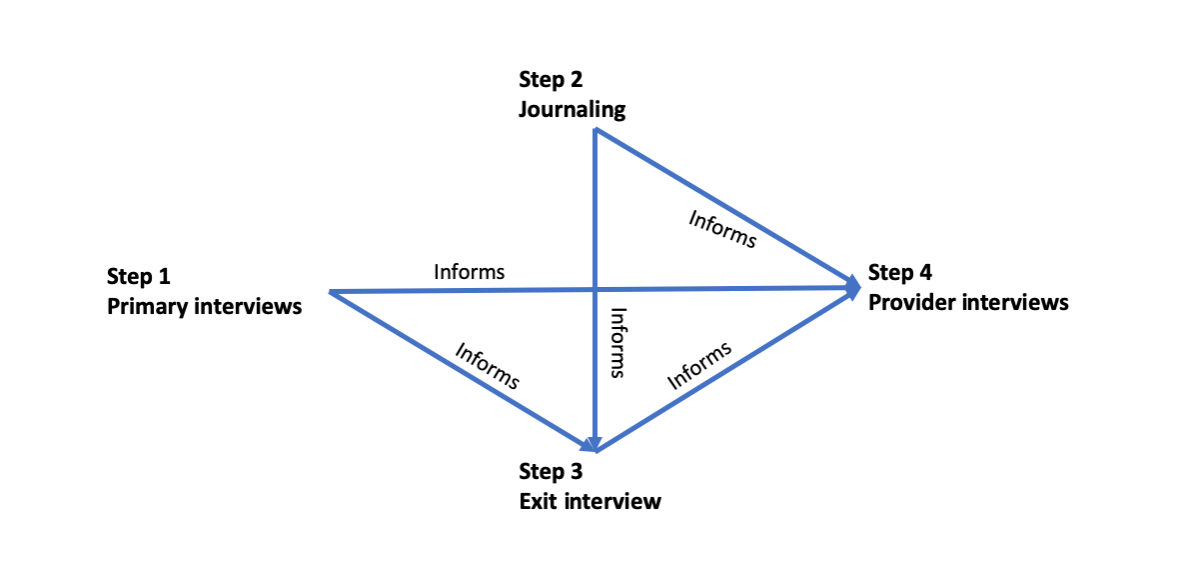
Data collection tools.

Data were collected (Textbox 1) first from primary interviews with patients, followed by patient journaling on tablet computers and exit interviews. We conducted provider interviews as the last step in data collection. Data from exit interviews with patients and provider interviews were collected to obtain both views. Figure 1 summarizes the data collection methods and how the different methods related to each other. Complete data (primary interviews, journaling, and exit interviews) were obtained from 90% (35/39) of patients (4 completed only the primary interviews). Four providers participated in the in-person interviews. Each patient interview (primary and exit) was conducted in person by the first (MO) and third authors (KH). These interviews occurred in a private meeting room in the College of Nursing, which is in the same campus as the anticoagulation clinic.

**Steps in data collection and information elicited.**

Step 1: Primary interviews (patient data collected)

- Care received by provider
- Social & physical environment
- Health-related activities
- Tools and technologies used
- Challenges and facilitators carrying out activities
- Interaction between clinical and daily activities

Step 2: Journaling (patient data collected)

- Health-related activities
- People involved
- Social, cultural, and physical contexts in home

Step 3: Exit interviews (patient data collected)

- Further exploration of issues discussed in Steps 1 and 2

Step 4: Provider interviews (provider data collected)

- Evaluation of health-related activities
- Description of clinical processes, policies, and development of therapy plan
- Provider

Those conducting the interviews used a predetermined list of questions; however, participants were also asked follow-up questions for clarification and to gain additional insights. *Primary patient interviews* provided information related to: (1) the care they received in the anticoagulation clinic; (2) their social, cultural, organizational, and physical environment; (3) daily health-related activities; (4) tools and technology they used to support these activities; (5) challenges and facilitators while conducting these activities; and (6) manner in which clinic-based health-related activities shape or influence activities in daily living contexts and health-related activities within daily life inform activities in the clinical context. *Journaling* provided data about health-related activities, the people involved in the activities, the temporal organization of the activities at home and other daily living settings, and the social, organizational, cultural, and physical contexts in which the activities occurred. Journaling also partially mitigated the recall bias. Journaling was accomplished by a tablet application that made voice entry possible. The application also moved all audio recordings to secure servers to facilitate data collection and analysis. Patients were provided approximately 20 min of training on the use of the tablet and the app. During the training, participants were instructed on how the journaling app worked, and the researcher reviewed the 11 guiding questions with participants. Participants were asked to journal at least every 2 days for a month and provided the telephone number of the research line in the event they needed assistance. Patients were given an option to keep the tablet computer as a thank for participating. *Exit interviews* were conducted with the patients after they completed journaling and explored issues that had been mentioned in the primary interview and journals. Exit interviews (Step 3) were conducted in person in the same room as primary interviews, with information used for triangulation. Patient primary and exit interviews averaged 48 and 20 min, respectively. *Provider (pharmacist) interviews* (n=4) lasted approximately 73 min. During this time, health-related daily living activities reported by the patients were explored and a description of the clinical processes, clinical policies, development of therapy plans, and provider perspectives dealing with challenges related to adherence of the patient.

We received consent from 39 patients for the primary patient interviews, journaling, and exit interviews and received consent from the pharmacists for provider interviews. For all of the data collection, any reference to a personal identifier (eg, names of people, organization, etc) was removed.

### Data Analysis

Interviews were transcribed verbatim professionally. The journal entries were reviewed and transcribed by KH. Qualitative analysis was based on a theory-driven approach [42] and the framework method of analysis by Gale et al [43]. The main concepts of the *Infinicare* framework [4] provided *a priori* codes, with additional codes identified inductively when textual elements did not fit within these predetermined codes [44,45]. The advantage of using an overarching framework was to increase the efficiency of the data analysis process and to build on prior studies. The advantage of deriving additional codes based on collected data allowed for better integration of the participant’s experiences.

Data from the 4 collection methods were coded, main themes were identified, and relationships between the main themes were examined. Data were managed using DEDOOSE 5.1.18. The analysis included independent coding of approximately 10% of the data by MO and KH. During this coding, various code ideas were discussed in light of the broader study purpose, such as (1) the process and contextual components associated with care for anticoagulation management and (2) the potential disconnect between health-related activities in the clinical setting and outside the clinical setting. Coding was then reviewed by 3 team members (MO, KH, and RV) until consensus was reached, following which a codebook was developed [46]. The codebook included codes, the concepts that were represented by the codes, specifications (definition, inclusion, and exclusions as applicable), and examples. We also allowed simultaneous coding, meaning that multiple codes could be applied to a piece of text [47]. KH applied the codebook to the remainder of the data. MO then examined embedding the codes within the overarching categories, based on their content.

## Results

### Description of the Participants

Study participants ranged in age from 25 to 83 years and had been receiving anticoagulation therapy for 2 weeks to 26 years (Table 1). Most participants were women and White. Educational attainment varied, although most had a minimum education of a college or associate degree (30/39, 76%). Most participants lived with someone. The median household income was US $60,000; 3 of the 4 providers had a PharmD degree, the other had a BS degree. The average experience of the providers was 15 year.

The gaps are the operationalizable aspect of the *Infinicare* framework and can be more directly used to guide HIT development. Our analysis revealed 3 aspects of gaps: reasons, consequences, and strategies to overcome these gaps (Textbox 2).

**Table 1:** Patient participant demographics from anticoagulation clinic (N=39).

Demographics		Values
**Age (years)**		
	Median	56
	Range	25-83
	Mean (SD)	53 (16)
**Therapy duration**		
	Median (years)	3
	Range	2 weeks to 26 years
**Gender, n (%)**		
	Female	23 (59)
**Ethnicity, n (%)**		
	White	27 (69)
	African American	9 (23)
**Highest education degree, n (%)**		
	Masters	7 (18)
	College	8 (21)
	Some college or associate degree	15 (38)
	High school	5 (13)
	Less than high school	4 (10)
**Living situation, n (%)**		
	Alone	6 (15)
	Spouse only	12 (31)
	Spouse and ≥1 child	7 (18)
	Family members or parents	9 (23)
	Roommates	4 (10)
	Other	1 (3)
**Income (US $)**		
	Median	60,000

**Reasons, consequences, and strategies used to overcome gaps.**

Reasons for Gaps

- Recommendations did not fit into patient’s daily routine
- Recommendations did not fit living context
- Information not transferred across settings

Consequences

- Cognitive and physical workload on the patient
- Poor patient satisfaction
- Compromised adherence to therapy plan

Strategies

- Patient-generated strategies
- Routines
- Collaborative therapy management
- Social environment
- Tools and technologies

### Reasons for Gaps

The reasons for gaps were from 3 areas related to therapy recommendations: misfit with the patient’s daily routine, misfit within the context of daily living, and information was not transferred across settings.

#### Recommendations Did Not Fit Into Patients’ Daily Routine

Daily routine is a structured, temporal cycle of activities that occurs daily or in some instances weekly. Participants noted difficulty integrating new activities (eg, cognitive remembering to take medication and physical-going to appointments) into their existing personal routines. These new activities competed with other activities in terms of time, money, and attention (Table 2, Quote ID #1 [ID 1]). Having a busy schedule (multiple jobs and family-related responsibilities) further worsened the challenge and required devoting additional time to planning the day. This was particularly emphasized by participants as an issue in planning meals (ie, deciding what to cook or shop for) and monitoring food consumption. Participants reported changes from previous meal-planning routines or grocery shopping norms. However, new routines may not be integrated and adapted quickly. Those with comorbidities requiring multiple therapies have magnified this challenge. In the words of one participant, additional therapy-related activities to an already busy schedule “slows [them] down.” Activities necessitated by therapy recommendations only fit into strict daily routines. Weekends, travel, seasonal, or religious events made adherence difficult (ID 2).

Therapy recommendations were not easily integrated into social and extracurricular routines. Food choices influence the effectiveness of the anticoagulant, requiring changes to dietary habits not only for participants but also for their families. Alcohol consumption has a strong influence on dosing. As alcohol also serves as a social activity, therapy recommendations necessitated changes in social routines such as avoiding social situations where drinking occurred. Participants also avoided social engagements to evade potential questions about their health (ID 3). Finally, due to the potential for serious bleeding from trauma (a side effect of anticoagulation), participants reported relinquishing some hobbies (eg, mountain biking; ID 4) or work-related activities (eg, riding horses).

**Table 2:** Quotations supporting selection of categories and subcategories.

Category, subcategories, and quote ID			Sample quotes	Source of quotes
**Reasons for gaps**				
	**Therapy recommendations do not fit into daily routine (additional cognitive and physical steps; busy life schedule is a challenge; planning challenges; comorbidity; variation in routines; decreased social engagement; giving up some hobbies)**			
		1	“It's nearly 8 o'clock and I'm just now getting home I left the house at 6:30 this morning. I didn't pack a lunch and who knows what I ate in terms of Vitamin K and now I have to go back to remember try to figure out what I'm having for dinner, and I threw up because I'm still sick so that'll be fun to try to guess again today” (additional cognitive and physical steps; busy life schedule is a challenge; comorbidity)	Journaling
		2	“I forgot to take my medication this morning at the normal time. Since I have the day off and the next day off with the holiday, I am on a little different schedule so I am going to have to be a little more conscious that I don’t start my day without taking my medication like I did today.” (planning changes; variation in routines)	Journaling
		3	“Went to a new neighbors house they didn't realize that I couldn't drink. [I] didn't want to explain it so just said that I was designated driver even though we were all just walking home, but I hate having to explain” (decreased social engagement)	Journaling
		4	“... knowing I'm on Warfarin...they don't want you to get lightheaded or anything like that. So I can't jog like I want to” (giving up some hobbies)	Primary patient interview
	**Therapy recommendations do not fit into patients’ living contexts (social; organizational; physical; cultural; temporal)**			
		5	“My husband wants to have a variety in the diet. I really can’t do that. I have to keep my vitamin K levels consistent. That’s the reason I tend to eat a lot of the same foods.” (social)	Journaling
		6	“Sometimes I get frustrated because I need afternoon appointments, period. I need afternoon appointments and I have to try to get them on Thursdays as much as possible, because that is the only time transportation is available.” (organizational)	Primary patient interview
		7	“It is one bedroom, so you don't get any privacy and that is a little bit difficult.” (physical)	Primary patient interview
		8	“I know my highest INR^a^ was 3.9 and it was after my stepdad passed away I hadn't been eating salads. I was back in Michigan for 10 days. Hadn't really been eating healthy. And definitely taking in more alcohol. That was kind of a scary thing. When you know that, it is wow! The possibility of if you had an accident and bleeding more would be greater.” (social)	Primary patient interview
		9	“There's been so much going on because of Christmas and New Years and my son's birthday. Which was hard for me, because with Christmas and shopping and everything else that was going on during the last month, it was a little difficult to stay on task.” (cultural; temporal)	Patient exit interview
	**Necessary information is not transferred across settings (insufficient transfer from the daily living to clinical settings; insufficient transfer from clinical settings to the daily living)**			
		10	“I usually lie to [providers]. If they say have you missed a dose I say no. Cause it is just easier than saying well, actually 3 weeks ago I did. Or ...I figure by then it doesn't really make a difference. I'd probably tell them if it was within 48 hours that I missed a dose and my bloodwork was off. Then I'd probably confess to it.” (insufficient transfer from the daily living to clinical settings)	Primary patient interview
		11	“A lot of challenges are misconceptions. Often patients will say, I can't take this. I don't like taking Warfarin because I can't do activities. I'm afraid I'm going to fall. I'm going to hit my head and have a fatal bleed. Whereas in reality, with Warfarin, you shouldn't let the medicine control your lifestyle... But we tell people to be safe.” (insufficient transfer from clinical settings to the daily living)	Provider interview
**Consequences**				
	**Additional cognitive and physical patient workload (remembering; decision making; physical workload)**			
		12	“the biggest one is probably just remembering to take [The medication]. Before being diagnosed with this, I never had anything that I had to take every single day.” (remembering)	Primary patient interview
		13	“I was having problems managing taking my...Antibiotics! 2 hours before taking my other meds. My anticoag. the decision was either I skip the antibiotics, or I skip the anticoag. So I skipped the antibiotics. But that seemed to happen like 4 times this last month.” (decision making)	Patient exit interview
		14	“And that is still a struggle with just being busy. And then also, in that, I have to kind of watch. If I know I'm going to be out of town, then I need to make sure not to run out of my prescription. To make sure I get things refilled and just being more strategic with the pharmacy and making sure I have refills and that I get there before I leave, if I'm going somewhere or something like that.” (physical workload)	Primary patient interview
	**Poor patient satisfaction (difficulties and inconvenience; frustration; overwhelmed)**			
		15	“The best is definitely with the menstrual cycle. That is just something that is in my family. And it is just very, very heavy within our family. Heavy cycles. And if I continue to take the Coumadin during that time, for like 2 of the days, I literally cannot leave the house. It is just too heavy. So I have to stop during that time. And the previous providers just did not understand that.” (difficulties and inconvenience; frustration)	Primary patient interview
		16	“It is getting better, but it is almost more of an annoyance at this point. That it is always there. That it is always part of what I have to think about. But it is just kind of become like your multiplication facts. You've memorized it. It's there. It is just part of memory space, at this point.” (frustration; overwhelmed)	Patient exit interview
	**Compromised adherence to therapy plan (unintentional; by choice)**			
		17	“I forgot to fill my weekly pill tray. And so tonight I took the wrong dose and so I need to fill it up with the right dose.” (unintentional)	Journaling
		18	“Accepting the fact that sometimes I try to sneak like cranberry juice or grapefruit, like when no one is paying attention.” (by choice)	Primary patient interview
**Strategies**				
	**Patient-generated strategies (self-adjusting the dose; green-alcohol balancing; timing of medication; remembering mechanisms)**			
		19	“After dealing with [bleeding nose] for years I finally said I'm not doing it this way anymore and I started changing my own dosing, according to my symptoms.” (self-adjusting the dose)	Patient exit interview
		20	“And when I was drinking, I was drinking, you know, quite a bit. Like daily almost. (laughing) And yeah, I was real cognizant of making sure I took in even more greens to counteract the alcohol” (green-alcohol balancing)	Primary patient interview
		21	“Why ‘The Price is Right?’ Because it is something that helps me...I just woke up one day and I took it and the ‘Price Is Right’ was on. And then the next day I saw, oh well, the Price is Right is on, it's time to take it.” (timing of medication; remembering mechanisms)	Primary patient interview
	**Routines (medication use; food consumption)**			
		22	“I take my Coumadin usually at 4:30 every afternoon. And that's when we feed the dogs. So it is kind of a routine. And when we are on the road, we do the same thing. The dogs have to be fed at 4:30. There ain't no ifs, ands or buts.” (medication use)	Primary patient interview
		23	“I guess [providers] got it to be my routine. [providers] figured out what I need. And I tell them ok. I eat Pizza on Wed. Thurs. I'm going to have my Spinach. Friday I'm going to have chicken. Then I make soup. And you know? And then I pretty much...And I got on a new thing where I make Shrimp Louie. I try to have Shrimp Louie every week or two. And you know...So I guess my diet doesn't change a whole lot. Old Irish guy. Meat and potatoes. So that is kind of where I think it makes it easy for me. I guess I'm kind of mundane and routine.” (food consumption)	Primary patient interview
	**Collaborative therapy management (patients being involved; plans around patients’ life; shared decision making; establishing trust)**			
		24	“Every time I have questions about something like, when winter is coming and I know I am going to be eating less fresh greens or something, [clinicians] are willing to say ok, well we can proactively start to cut back your Warfarin if you think you are going to be eating less and then see how you are doing.” (patients being involved; plans around patients’ life; shared decision making)	Patient exit interview
		25	“I write down a note to myself like, typically I would say, [this patient] eats greens two to three times a week. But his INR was low because he had collard greens over Thanksgiving and that made his INR low. So I know I'll give him a little extra and then we'll go back to what he was doing before.” (plans around patients’ life)	Provider interview
		26	“So once you start to get to know [patients], you kind of get to know how aggressive you can be with making adjustments and how conservative you should be.” (plans around patients’ life)	Provider interview
		27	“In terms of my relationship with [the provider] as far as trust goes. I trust her recommendation when she does or does not adjust my dosage. It makes me feel more comfortable with her to ask questions. I think it makes a huge difference. And also in my willingness to keep coming back.” (patients being involved; establishing trust)	Primary patient interview
	**Social environment (emotional support; experience sharing; protecting risks; make suggestions; transportation; reminder; prepared food)**			
		28	“I got an ear full of people. My girl, my mom, my sister. So support is there, to the point of where I'm going to do what I have to do. Cause I'm not going to put up with these people. So it's there and they are real concerned and making sure I'm taking care of myself and on my medication, like I'm supposed to. I got a very good support base” (emotional support)	Primary patient interview
		29	“My mom has been on it since she was 16, so I've learned so much from my mom.” (experience sharing)	Patient exit interview
		30	“Collaterally, they [friends] help. Like if I need to do some chores or heavy lifting, or something that puts me at risk for getting bruised or beat up, and they are there, then they will usually jump in and give me a hand.” (protecting risks)	Patient exit interview
		31	“The lady at my job, who had the stroke from the blood clot - she is still alive. She told me to do Yoga. She said Yoga is a good substitution that will help replace jogging.” (make suggestions)	Primary patient interview
		32	“I try to schedule on Monday's now so that my fiancé' can drive me, cause he doesn't work on Monday's now. So he will drive me and then it's not that big of a deal.” (transportation)	Primary patient interview
		33	“My wife is always reminding me probably about every night. So she stays on top of it.” (reminder)	Patient exit interview
		34	“[roommates] contribute a great deal, they cook certain things to make sure I can eat it.” (prepared food)	Journaling
	**Tools and technologies (tools that patients bring in; tools that clinic provides; INR machines)**			
		35	“Really the only challenge is remembering medicine and I use a pill container to get over that challenge.” (Tools that patients bring in)	Journaling
		36	“[Providers] give me calendars where I can write it down. And I cross it off when I take it. And when I don't take it, they know when it's blank I didn't take the medication.” (tools that clinic provides)	Patient exit interview
		37	I'd go back to being able to do the INR at home. (INR machines)	Primary patient interview

^a^INR: international normalized ratio.

#### Recommendations Did Not Fit Into Patients’ Daily Living Contexts

We distinguished the living context from daily routines. Living context is a manifestation of the characteristics of settings that serve as obstacles or facilitators to performing routines. For example, the social context highlights pressure and encouragement from others rather than the fact that having a routine that involves a social life that is inherently challenging.

Participants reported misfits of anticoagulant therapy within their social, organizational, physical, cultural, and temporal contexts. Their social environment could adversely influence their consumption of foods, resulting in conflict with their therapy (ID 5). Misfits related to the organizational context included lack of access to transportation to the clinic and limited clinic hours and their work schedule (ID-6). Challenges related to the physical context related to limited residential space, resulting in a lack of privacy and need to spend more time outside or away from home (ID 7). Clinicians reported that cultural activities such as fasting practices for religious purposes and excess consumption of green tea could be problematic. Participants reported that the temporal context (eg, Thanksgiving, birthdays) came with new requirements that made therapy management difficult (ID 8 and 9).

#### Information Not Transferred Across Settings

Insufficient transfer of information from daily living to clinical settings was represented by incomplete or inaccurate patient history (ID 10). This occurred through misconception by patients regarding therapy, not recalling the therapy plan, and/or misinterpreting the provider’s comments. Rushed encounters, patient misconceptions, and organizational policies (such as not accepting patients late for an appointment or lack of available timely appointments) could lead to insufficient transfer of information from clinical to daily living settings (ID 11). Missing an appointment means there is no transfer of information in either direction, resulting in an inability to create a plan designed specifically to suit the patient’s lifestyle.

### Consequences of Gaps

We grouped the consequences of gaps into 3 themes: cognitive and physical workload on the patient, poor patient satisfaction, and compromised adherence to the therapy plan.

#### Cognitive and Physical Workload

Cognitive workload issues included additional remembering and decision making. Examples include the type and quantity of recent food consumption, taking medication at the correct time and dosage, obtaining medications from the pharmacy, and making appointments (ID 12).

Additional decision-making tasks were decisions during grocery shopping, cooking, plate portions, and ordering at restaurants to ensure that vitamin K consumption was consistent. Almost all participants took warfarin daily, but the daily dosage could vary with the day of the week. On occasion, a decision had to be made on whether to skip a dose if the medication was not taken as scheduled (ID 13). Participants faced implicit decisions about whether to focus on adhering to therapy versus engaging in activities that may potentially jeopardize patient outcomes. Physical workload issues included challenges related to attending appointments, obtaining medications from a pharmacy, and traveling back home due to forgetting medication (ID 14).

Analysis highlighted that some causal links (anecdotal level) between the reasons and consequences of gaps are related. ID 12 exemplifies the additional cognitive workload on patients, when additional steps are not well integrated into daily living routines, and possibly the need for preplanning. Busy lifestyles exacerbate this misfit.

#### Poor Patient Satisfaction

Increased workload is a phenomenon in which patients have an emotional response that can affect health-related quality of life. This emotional response falls into the theme of satisfaction. For example, dealing with bleeding was reported as a burden on physical workload; however, the emotions of frustration, fear, and shame (as it relates to stigma) would fit well under satisfaction. Participants reported a wide range of negative emotions due to the challenging experiences resulting from the gaps reported above. One participant reported excessive bleeding during her menstrual cycle, making it difficult for her to leave home, which led to frustration and inconvenience (ID 15).

Other reported frustrations included food constraints, the need for food tracking or remembering, and renewing prescriptions. Some participants reported a social stigma and embarrassment: “talking about anticoagulation makes [them] feel old.”

The therapy requirements could be overwhelming. The therapy could be an annoyance since “It is always a part of [the participant’s] life.”

#### Compromised Adherence to Therapy Plan

Adherence was not a problem for all patients; however, reduced adherence could lead to serious health consequences. Participants reported the following adherence related issues: delay in taking medication, taking a wrong dose, missing a dose, and not following the dietary restrictions (ID 17).

Some instances of lack of adherence were by choice. Some reported “sneaking in” contraindicated foods (ID 18), consuming excess alcohol at special events, and missing medication when away from home. Adherence could be due to misfit of the therapy plan with the patient’s daily routines (ID 2) or the context of their daily living.

### Dealing With Gaps

Five strategies were used to cope with the challenges of gaps: (1) patient-generated strategies, (2) routines, (3) collaborative therapy management, (4) social environment, and (5) tools and technologies.

#### Patient-Generated Strategies

We defined patient-generated strategies as improvisations (not necessarily approved by their clinician) by patients with the intention of better self-management. These strategies included avoiding adverse events (bleeding) and helping with remembering. Strategies were typically discovered by patients over time and by *trial and error*.

One common strategy was self-adjusting the medication dose to avoid bleeding and symptoms (ID 15 and 19). Some reported the consumption of greens when they had consumed alcohol, under the assumption that greens balanced out the effects of alcohol (ID 20). Other reported patient-generated strategies included improvising remembering mechanisms, training the dog to bring the phone, and carrying extra medications (ID 21).

These strategies were not necessarily suggested by clinicians, but clinicians were often aware of their use. Clinicians reported mixed opinions about the effectiveness or harm of these strategies.

#### Routines

Routines were defined as repeating the same set of activities at approximately the same time of the day. Participants utilized routines for cognitive support (mostly remembering). For example, medication use was timed by daily events (feeding dogs, a TV show, and taking a shower) or a specific time of day (ID 22). They also used weekly events or times to fill their pillbox. Grouping and placing medications in different areas of the home was another way of routinizing medication use. Routines regarding food consumption included frequenting the same restaurant that provided the same food and having a fixed weekly menu (ID 23).

The majority suggested that establishing routines helped with therapy management; however, busy periods made the following routines more difficult. New routines were easier to follow if they fit into their existing daily routines. Participants reported breaks in routine during holidays.

Clinicians reported that routines were one of the main strategies they suggested; however, the risk of establishing routines was difficult to *alter* with changes in therapy. Summarized by one pharmacist as, “habits are hard to form and hard to break.”

#### Collaborative Therapy Management

Collaborative therapy management (CTM) was defined as active efforts by patients and clinicians (and health care system with the available resources and opportunities to the patient) to work together for optimal health outcomes. Participants contributed to CTM by providing an accurate history, being involved in decision making and engaging actively in their health (eg, prioritizing health-related activities and taking responsibilities, ID 24). Clinicians contributed to CTM by asking personalized questions, developing therapy plans around the patient’s life, making it easy to comprehend and apply, encouraging engagement, and providing tools that allow patients to take responsibility for their health (ID 25).

Therapy plans required knowing the patient’s lifestyle, individualizing recommendations congruent with the preferences and routines of the patient, and adjusting dosages around the patient’s daily life (ID 26). Making the therapy easy included being flexible in appointment times, expanding office hours, having processes that allowed patients to use local laboratories, and accommodating transportation limitations.

CTM also included shared decision making and iteratively adjusting therapy until a solution that was mutually agreed upon was reached. Both clinicians and patients highlighted that establishing a trusting relationship was a prerequisite for successful CTM (ID 27). CTM is particularly important for patients with complex needs due to multiple comorbidities and living situations. In particular, CTM highlighted difficulties in using the strategies described.

#### Social Environment

The patients’ social environment varied and included: family (mother, sister, grandchildren, aunt, and in-laws), friends (social media as well as in person), colleagues, partners, pastors, and roommates. The social environment provided emotional support by not eating restricted food in front of the patient, looking out, being mindful, encouraging, sharing funny material through social media, showing understanding, and “checking-in” periodically (ID 28). The social environment goes beyond just providing emotional support but also logistical, cognitive, and informational support. Some found it helpful to have others convey their experience with the same therapy (ID 29), protection from risks (ID 30), made (food and exercise) suggestions (ID 31), provided transportation (to appointments and for medications; ID 32), reminded of medication use and food restrictions (ID 33), and prepared appropriate food (ID 34).

#### Tools and Technologies

The most commonly reported tools and technologies included a pillbox, reminder alarm on smartphones, and a calendar tool provided by the clinic (ID 35 and 36). This calendar displayed the days when the medications were received and skipped. Some reported their use of smartphone apps to track food consumption. Many reported their willingness to use INR machines at home; however, the clinic did not support it (ID 36).

These strategies help narrow the gap by reducing the effects of the 3 aforementioned reasons and mitigating the consequences. Routines and social environment can help with better fit of therapy plans, and CTM can mitigate patient dissatisfaction.

## Discussion

### Principal Findings

This study examined chronic disease–related challenges by focusing on gaps between therapy plan development (ie, clinic based) and implementation (ie, daily living based), using the case of anticoagulation therapy. Gaps were latent and not directly observable, but their effects were evident. We ensured the collection of rich data on gaps by employing multiple data collection methods that inform each other. We also collected data from both patients and providers. The use of a framework (ie, *Infinicare*) ensured that only relevant data were collected on all aspects of the gaps. The reasons for and consequences of gaps as well as strategies to overcome them were identified. The reasons for gaps can prompt needs assessments and inform systematic interventions (eg, informatics, policy, educational, operational) that narrow these gaps. The consequences should also be taken into account when designing and implementing interventions. Moreover, these consequences can serve as evaluation criteria for interventions. Interventions should be congruent with the 5 reported categories of strategies for bridging the gaps. The reported strategies are a jumping-off point, and other strategies may be developed.

Understanding the reasons for the consequences of and strategies to overcome gaps can also help integrate the concept of gap into the current and future conceptual models and frameworks of health management. These models and frameworks can better explain the factors that affect health management.

Previous studies [26,48] that focused on the individualization of health care services and the transition of care have highlighted some of the themes discussed in this paper, such as collaboration among clinicians, patients, and family members; social support; provider awareness and knowledge of patient situations; understanding patients’ cultural practices and beliefs; understanding places in which the patient lives; emphasis on the patient and family experience; focus on home and community-based care; emotional support and alleviation of fear and anxiety; and encouragement of continued social roles. Moreover, studies that focused on self-management activities and adherence work stressed the importance of daily living contexts [2], discrepancies between clinicians’ and patients’ perspectives on self-management [49], and localization of therapy management [20]. However, this study went beyond previous findings by identifying the *concept of the gap*. Conceptually, gaps can provide more insights to researchers by better explaining incongruence among work system elements [2] in chronic disease management. This study provides a foundation for collaborative HIT design guidelines by highlighting relationships among the precursors to, consequences of, and counter measures against cross-setting gaps.

### Design Implications

The 3 reasons identified for gaps underscored the need for clinician access to HIT that would incorporate more information about the patient’s daily living environment. Current consumer informatics technologies (eg, personal trackers, food log applications) support data collection on only a limited number of activities of daily living (eg, exercise, food consumption); however, these data are not always leveraged in developing therapy plans taking into consideration a patient’s routines and context [50,51]. This study shows that other types of routines (eg, social life) also need to be captured and accounted for. Moreover, there is a need to capture the interplay between different types of routines (eg, social life, food consumption, medication administration). The use of such rich data in creating therapy plans would require better integration of patient-generated data into EHRs. Clinical decision support systems that use machine learning algorithms can harness historical clinical and patient-generated data and assist in developing individualized therapy plans [52,53]. Mobile technologies provide opportunities to better capture essential information in each clinical and daily living setting and information that can then be retrieved as needed across settings [54-56]. We also contend that, instead of prioritizing either clinical or daily living settings, technologies should foster collaborative dialog. This collaborative dialog should include (1) joint clinician-patient interpretation of patient-generated data [39] and (2) discussion of patient-driven therapy plan translation options [20]. The reasons for gaps can guide clinicians’ question-asking and decision-making behavior. Focusing on gaps can help inform interventions with (1) technology, (2) policy, (3) educational, and (4) operational components that would support management in chronic disease. In fact, any collaborative HIT intervention that would narrow or close the gaps could need all 4 components. In addition, HIT designs can also be developed to promote sustained collaborative relationships between clinicians and patients [32,52]. There are no explicit guidelines on how to design and implement a truly collaborative HIT. This paper creates a starting point for the design and implementation of collaborative HIT. Design efforts should ensure that both clinicians and patients have tools and approaches for collecting, interpreting, and sharing information. Therefore, some of the potential next steps in designing and implementing collaborative HIT would be to create standard terminology for daily living activities (eg, food-related practice), developing machine-readable representations for daily living activities, and visualizations of these activities for both patients and providers.

Our analysis revealed patient-generated strategies for informing collaborative HIT design [17], but each strategy has attendant risks with the potential for unintended consequences. For this reason, these strategies should be vetted and implemented in consultation with clinicians, moreover because CTM (as we reported in the Results section) requires seamless, cross-setting information flow. HIT should be designed to be activated by patients and clinicians alike.

Owing to its outsized impact on patients’ decision-making processes, the social environment can both open and close gaps in chronic condition care and its maintenance. As a result, HIT should capture and communicate relevant social environmental information to clinicians to inform therapy plans [57,58].

Developing routines was reported as an effective strategy by both clinicians and patient participants. Today’s consumer technologies (eg, smartphones) partially support establishing routines; however, clinicians’ understanding of and contribution to these routines is limited, and some clinicians’ suggestions may not work for all individuals. Future collaborative HIT (eg, sensor-based technologies) should and can support clinicians’ understanding of patients’ existing routines and provide therapy plan options that can better fit in the context of patients’ home environments. Moreover, as both patients and clinicians report that routines are effective, new technologies can help generate routines to support health management in daily living settings. Collaborative HIT can play a role in identifying the reasons for the formation of gaps, monitoring the consequences of gaps, and supporting the implementation of gap-mitigating strategies.

An understanding of individuals’ daily living contexts can help providers better support patients’ chronic disease management by eliminating the 3 reasons for gap formation, as also highlighted by previous research [4,5,17,20]. Collaborative HIT can link currently disconnected clinical and consumer technologies to flesh out contextual factors [59] and attempt to neutralize potential discrepancies in social determinants of health.

The strength of this study is its theory-driven approach. The *Infinicare* framework informed data collection or analysis and focused the researchers on the cross-setting relationships among health-related activities. This study demonstrates the utility of *Infinicare* in both examining clinician-patient collaboration and informing collaborative HIT design and implementation. Moreover, this study extended the concept of the gap by providing a case showing how gaps can inform HIT design in the context of anticoagulation therapy. Another strength of this study is its use of multiple data collection methods. We used tablet-based journals complemented by interviews. Journaling allowed for moment-by-moment collection of critical data about patients’ health management activities, and interviews provided rich information about patients’ contexts.

### Limitations and Future Work

This study is limited in that it drew data from a single outpatient anticoagulation clinic that uses warfarin and does not have home monitoring (nonetheless, this is likely a typical US anticoagulation clinic). Although we captured rich information on the causes and consequences of gaps and strategies for avoiding them, other clinical settings may reveal additional gaps. In addition, the study design leaned toward the patient’s perspective rather than the clinician’s perspective (eg, discussion of gap-related consequences did not address clinician workload). It is likely that there are more consequences to clinicians (eg, in terms of workload, patient education) than we reported. In our future work, we will seek to better understand the gaps from a clinician’s perspective. To minimize researcher bias, we collected data from 3 resources and triangulated them across data sources. Data were collected and analyzed by multiple researchers.

### Conclusions

The study used multiple methods to explore the idea of gaps in a way to inform collaborative HIT. When designing and implementing these technologies, health-related activities conducted in daily living settings and clinical activities should be well connected and together they should be examined as a single workflow. Collaborative HIT can support clinicians and health systems to make decisions toward narrowing or eliminating the gaps can potentially improve patient and organizational outcomes.

## Abbreviations

CTM: collaborative therapy management
EHR: electronic health record
HIT: health information technology
INR: international normalized ratio
IT: information technology
